# Supplementary material for: Genomic epidemiology of SARS-CoV-2 in a UK university identifies dynamics of transmission
Source: Nat Commun. 2022 Feb 8;13:751. doi: 10.1038/s41467-021-27942-w (PMC8826310; doi:10.1038/s41467-021-27942-w)
Supplement: Supplementary file 1 — Supplementary Information [file 41467_2021_27942_MOESM1_ESM.pdf]

Genomic epidemiology of SARS-CoV-2 in a UK university identifies dynamics of transmission

Supplementary Figures

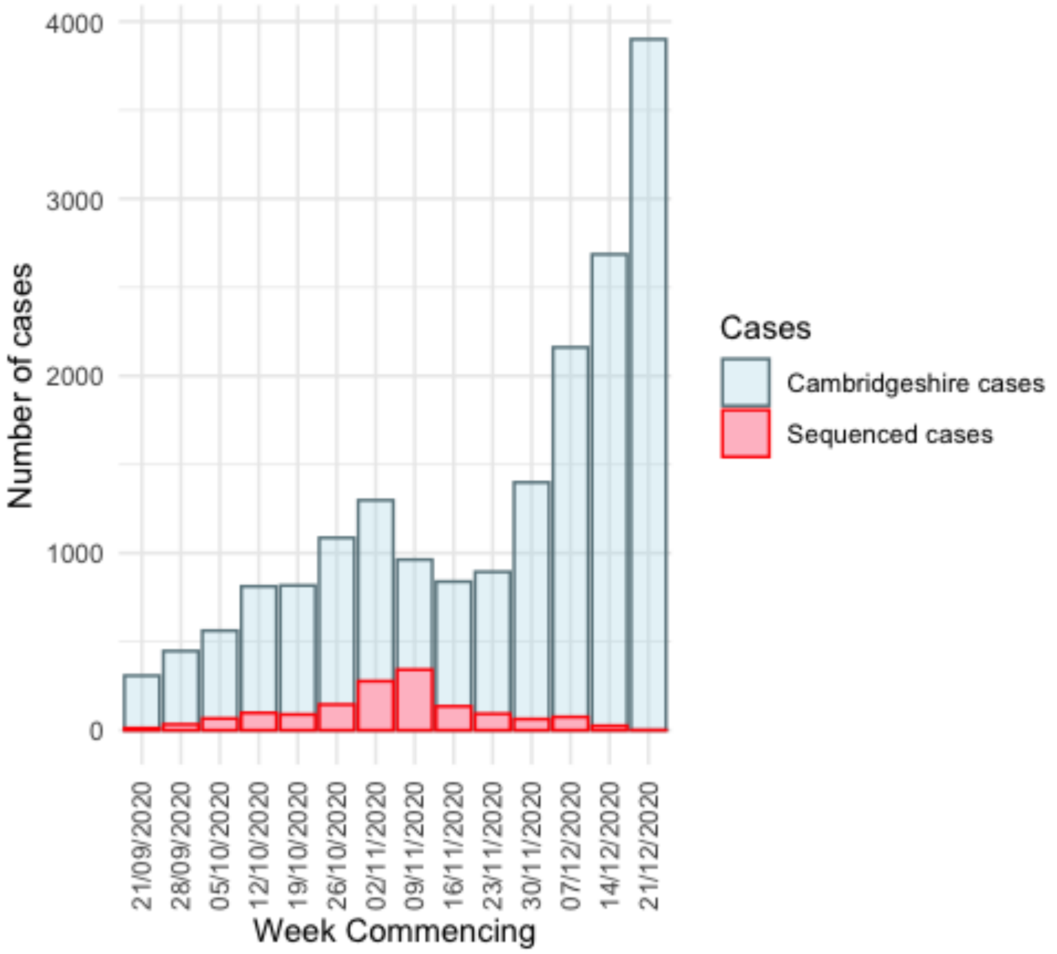

Supplementary Figure 1: Bar chart demonstrating the total number of Cambridgeshire cases including local community, university and hospital (light blue) and corresponding numbers of high-quality sequences available for the study. Source data are provided as a Source Data file.

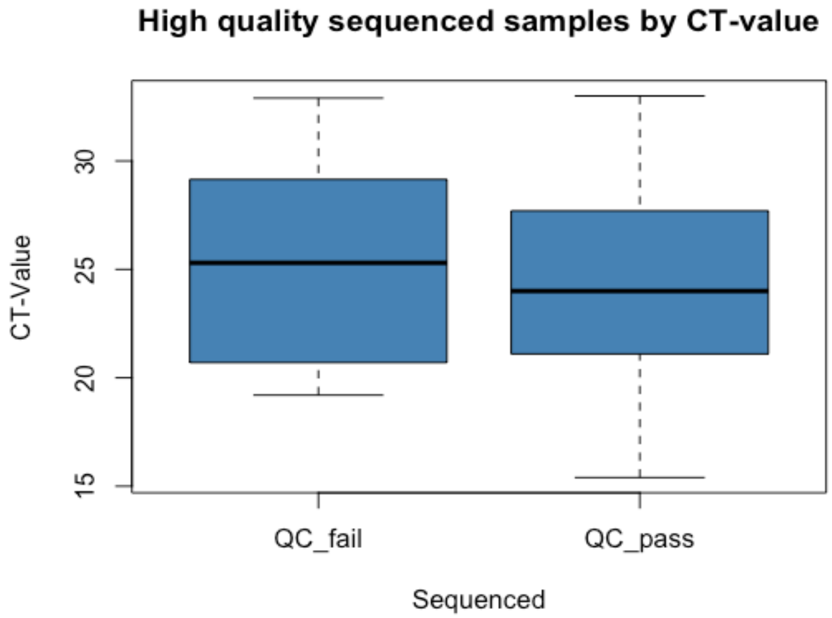

Supplementary Figure 2: Box plot to demonstrate difference in cycle threshold (Ct) values of those samples passing quality control (QC) thresholds to meet sequence inclusion criteria (<5% 'N' count and > 29000Kb file size) and those that did not. The difference between the Ct values of samples that passed QC versus those that did not was not significant (two-sample t-test, p = 0.27, n=468 biologically independent samples). The midline of the boxplot represents the median CT-value; the lower limit of the box represents the first quartile (25th percentile), and the upper limit of the box represents the third quartile (75th percentile); the whiskers (upper and lower) extend to the largest and smallest value from the box, no further than 1.5*IQR from the box. Source data are provided as a Source Data file.

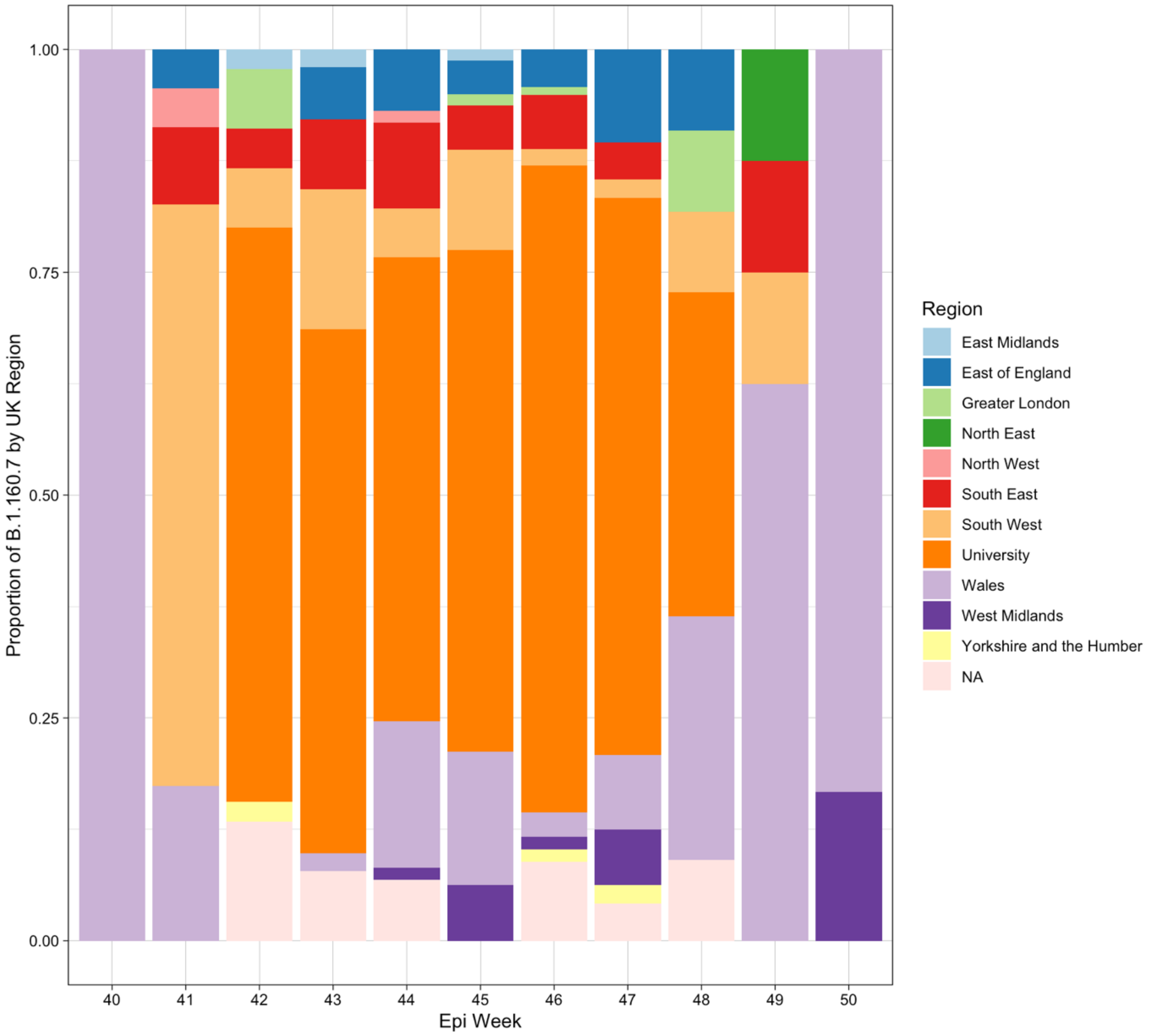

Supplementary Figure 3: Proportion of Lineage B.1.160.7 (to which cluster 1 belongs) sequences in each region of the UK. Regions are defined as 'Nomenclature of territorial units for statistics' (NUTS) regions, where the UK has 9 regions. Lineage B.1.160.7 was first sequenced in Wales, and then in the neighbouring South West of England, before the greatest proportion are found to be within the University of Cambridge. Cambridge is located within the East of England region. Source data are provided as a Source Data file.

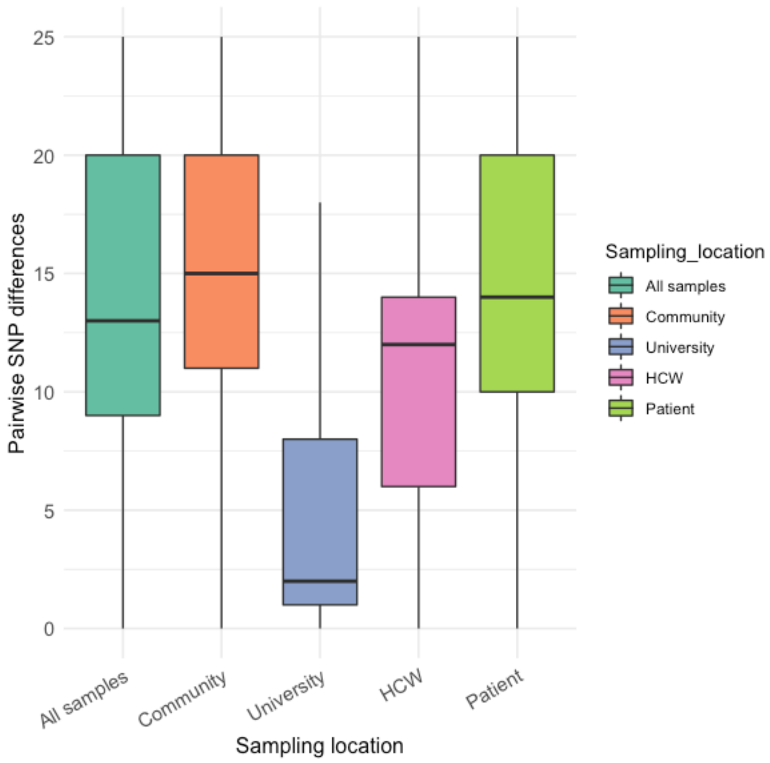

Supplementary Figure 4: The SNP difference among university students was much lower (two-sided Wilcoxon signed-rank test, p-value < 2.2e-16, n=1454 biologically independent samples) than among the rest of the Cambridgeshire community. This is likely to reflect the fact samples from University students were geographically and socially more closely related, and the establishment of fewer persistently transmitting lineages. The midline of the boxplot represents the median CT-value; the lower limit of the box represents the first quartile (25th percentile), and the upper limit of the box represents the third quartile (75th percentile); the whiskers (upper and lower) extend to the largest and smallest value from the box, no further than 1.5*IQR from the box. SNP = Single-nucleotide polymorphism; HCW = Healthcare Worker.

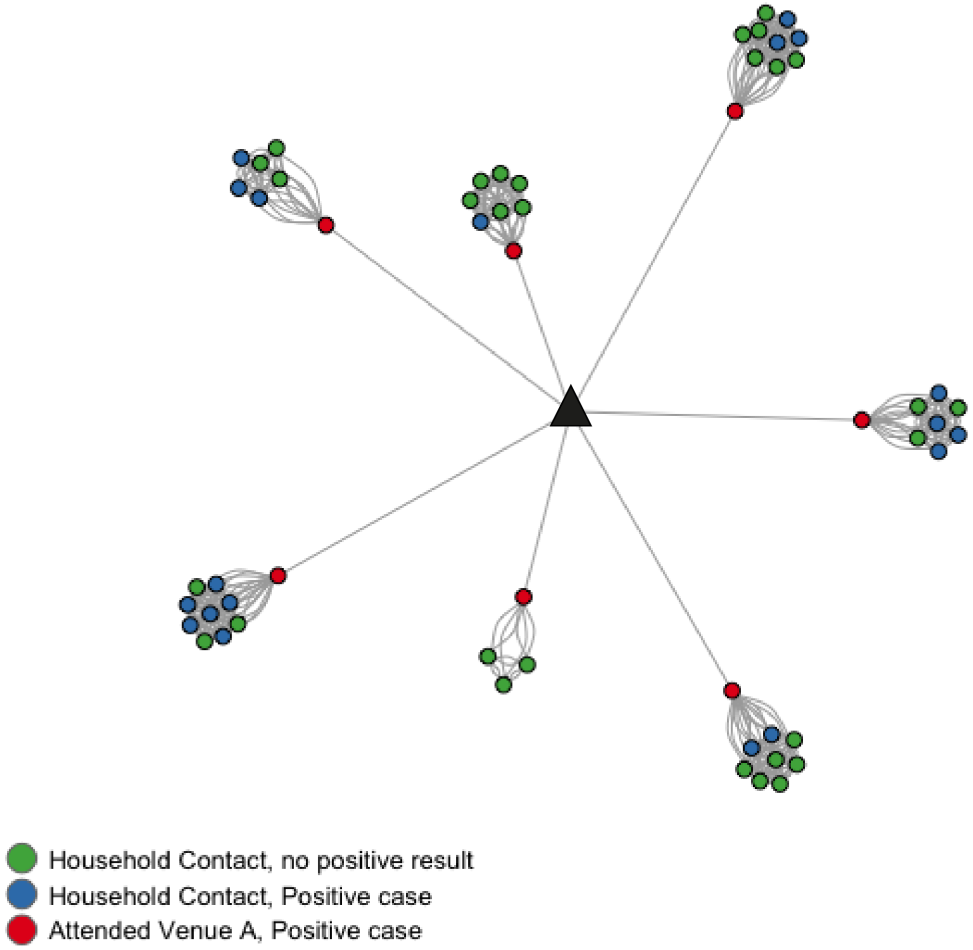

Supplementary Figure 5: Network diagram representing positive cases for SARS-CoV-2 within households of individuals attending Venue A in the first two weeks of the university term. Venue A (black triangle) was closely associated with cases in Cluster 1 and is strongly suspected to be important in dispersal through the university at the start of term. The network diagram represents all individuals who attended Venue A (black triangle) in the first two weeks of term and subsequently tested positive, as well as their household contacts (regardless of the availability of a SARS-CoV-2 genomic sequence) where available.

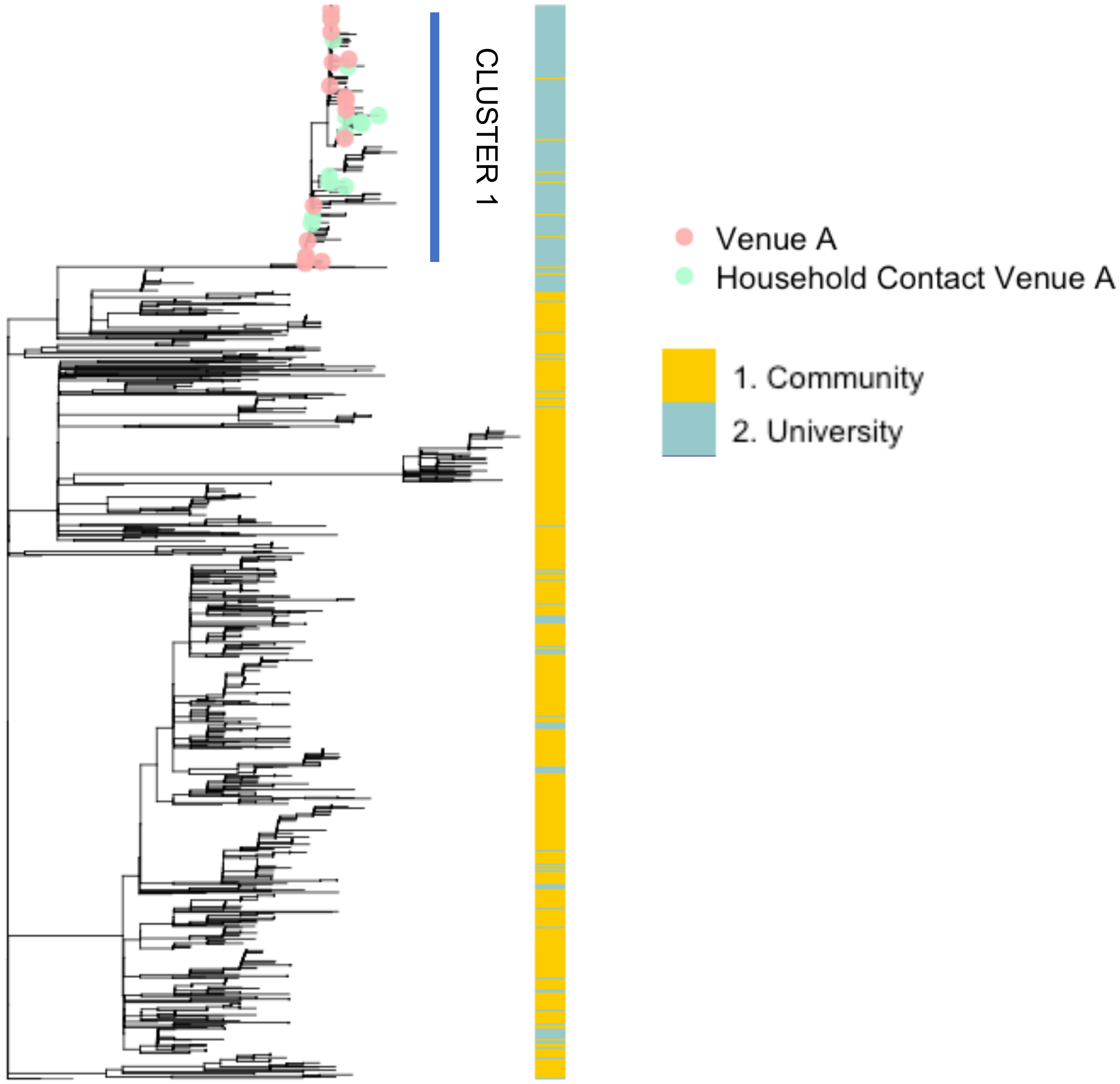

Supplementary Figure 6: Maximum Likelihood Tree of all isolates in the study demonstrating phylogenetic association of positive SARS-CoV-2 cases associated with Venue A. Venue A attendees (pink node leaves/tree tips) and household contacts of individuals who visited Venue A (green node leaves/tree tips) but were not sequenced are highlighted on the tree, located on Cluster 1. This venue was implicated as a possible source for the dispersion of SARS-CoV-2 across the university and increased transmission in the weeks around national lockdown. The vertical panel represents cases by location (general community and university affiliated members).

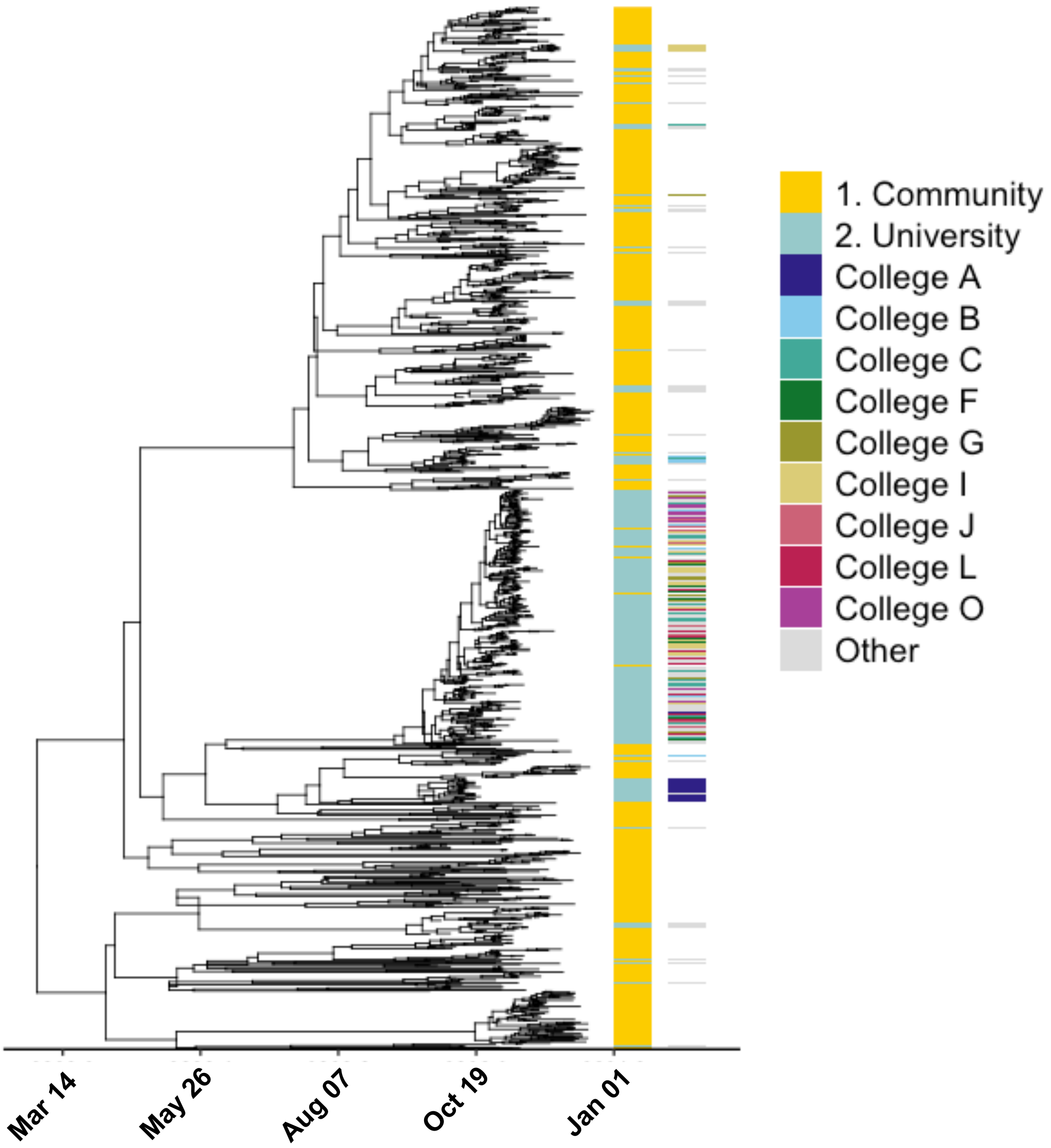

Supplementary Figure 7: Coalescent tree estimates of university and community cases with an exponential growth coalescent tree prior and a GTR+ Γ substitution model including all university and community high-quality genomes from the study period. A substitution rate fixed to $1x10^{-3}$ substitutions per site per year (s/s/y) was used under a strict clock model. Previous SARS-CoV-2 analysis have recommended a substitution rate in line with this[1,2] or that presented in the main text ($8x10^{-4}$ s/s/y). Of note, we can observe community and university cases remain segregated, with an epidemiological distinct university cluster (cluster1) that is divergent from its closest related cluster of Cambridgeshire community isolates (135 days (C.I. 102-169) prior to the start of term) demonstrating our conclusions are robust to changes in model parameters.

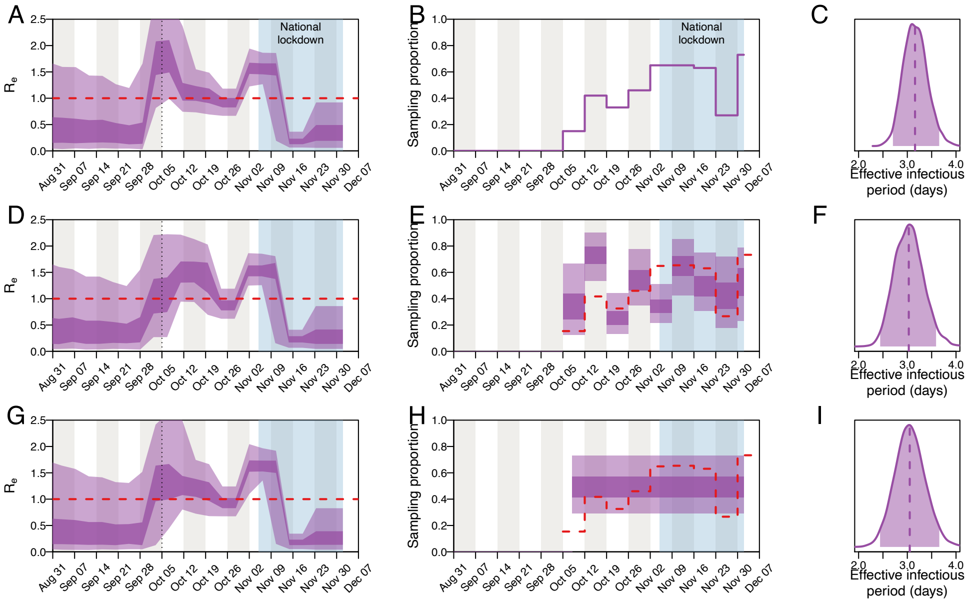

Supplementary Figure 8: Parameter estimates of cluster 1 with the birth-death skyline model when R_e is parameterised into 20 equidistantly spaced epochs, under different parameterisations of the sampling proportion (dark shading=50% HPD; light shading=95% HPD): (A-C) fixed to the empirical estimates (number of sequenced genomes from all University clusters divided by the number of positive tests among University staff and students), (D-F) fixed to 0 before the start of term and estimated for each week thereafter, and (G-I) fixed to 0 before the first week of term and assumed to be constant thereafter. See the caption of figure 5 for further details. R_e = Effective reproduction number.

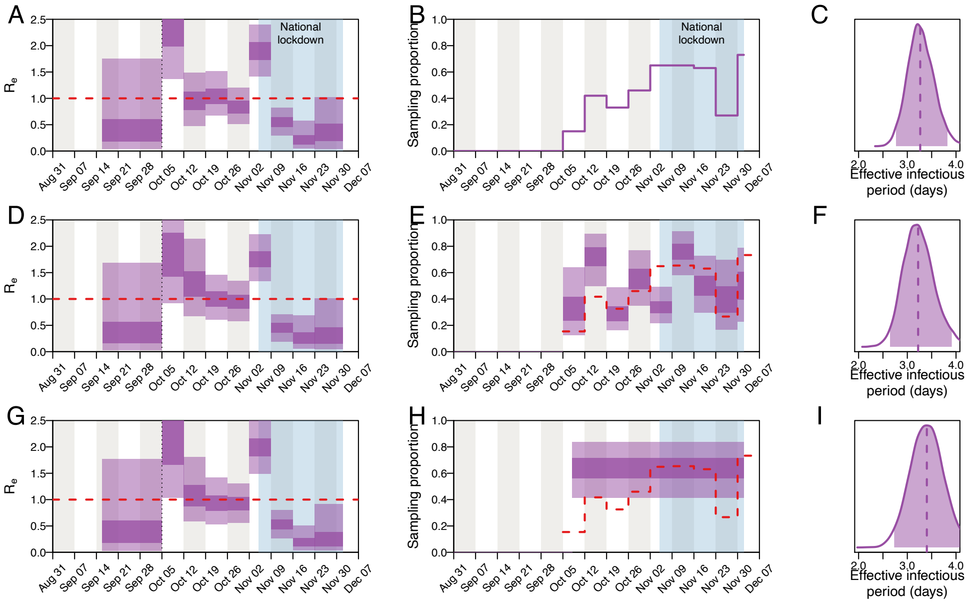

Supplementary Figure 9: Parameter estimates of cluster 1 with the birth-death skyline model when R_e is estimated for each week of term, under different parameterisations of the sampling proportion (dark shading=50% HPD; light shading=95% HPD): (A-C) fixed to the empirical estimates (number of sequenced genomes from all University clusters divided by

the number of positive tests among University staff and students), (D-F) fixed to 0 before the start of term and estimated for each week thereafter, and (G-I) fixed to 0 before the first week of term and assumed to be constant thereafter. See the caption of figure 5 for further details. R_e = Effective reproduction number.

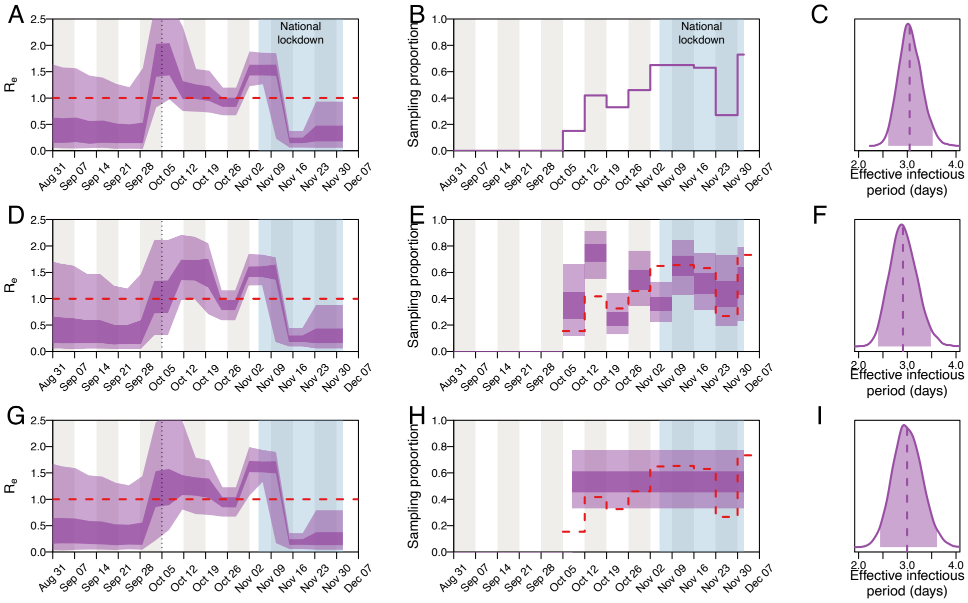

Supplementary Figure 10: Parameter estimates of cluster 1 with the birth-death skyline model when the clock rate prior is set to a lognormal distribution with mean 1x10^{-3} s/s/y (in real space) with standard deviation 0.1 and R_e is parameterised into 20 equidistantly spaced epochs, under different parameterisations of the sampling proportion (dark shading=50% HPD; light shading=95% HPD): (A-C) fixed to the empirical estimates (number of sequenced genomes from all university clusters divided by the number of positive tests among University staff and students), (D-F) fixed to 0 before the start of term and estimated for each week thereafter, and (G-I) fixed to 0 before the first week of term and assumed to be constant thereafter. See the caption of figure 5 for further details. R_e = Effective reproduction number.

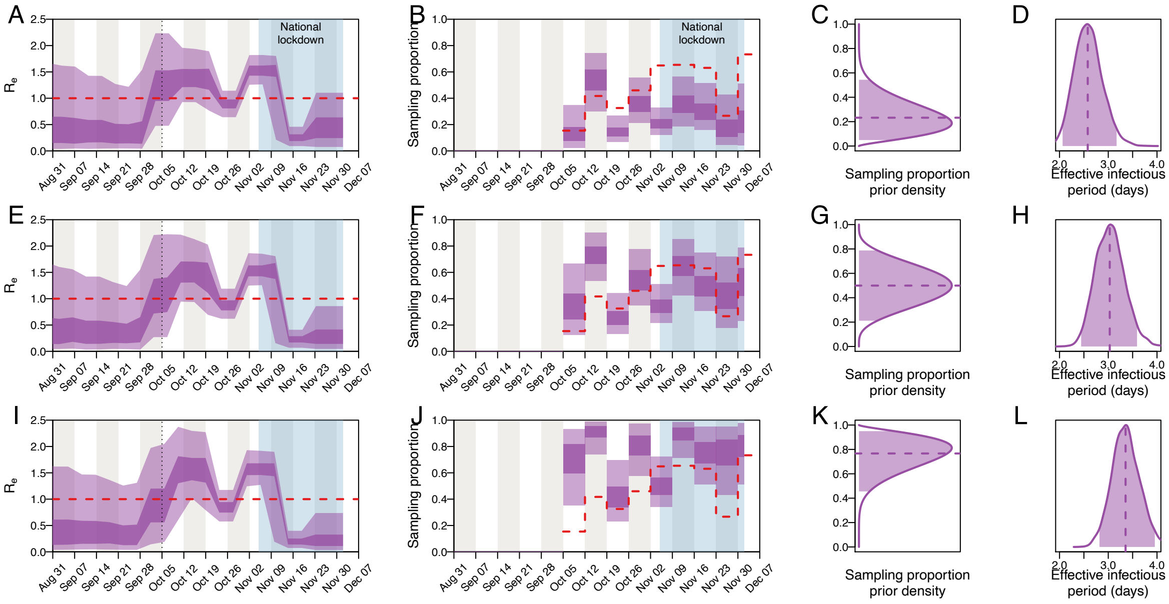

Supplementary Figure 11: Parameter estimates of cluster 1 with the birth-death skyline model when the sampling proportion prior is varied (dark shading=50% HPD; light shading=95% HPD): (A-D) Beta(2.5, 7.5), (E-H) Beta(5,5), and (I-L) Beta(7.5, 2.5). See the caption of figure 5 for further details. R_e = Effective reproduction number.

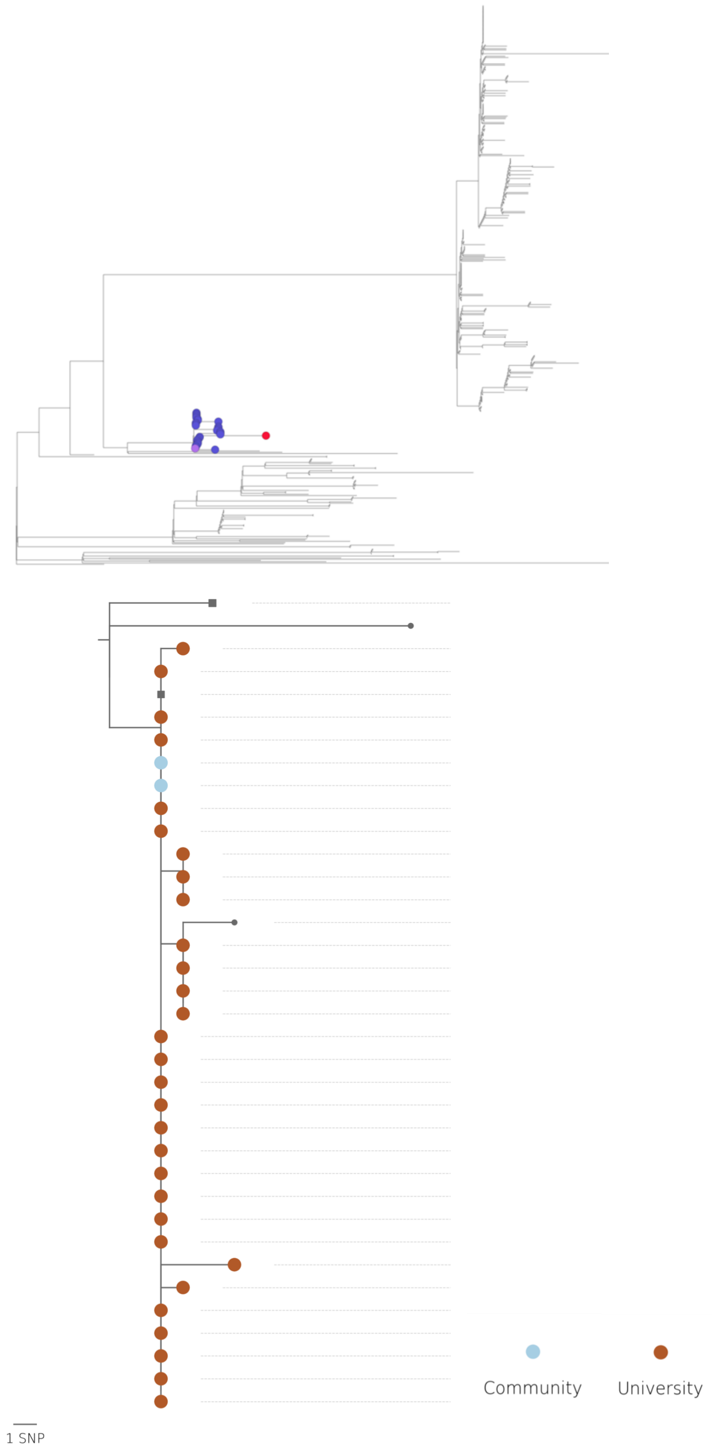

Supplementary Figure 12: Cluster 2 highlighted on a maximum likelihood tree of university cases, with associated CIVET cluster output demonstrating close phylogenetic relatedness. Sequences from GISAID used to contextualise study sequences by CIVET are represented by dark grey squares (collapsed nodes) and dark grey circles (individual sequences).

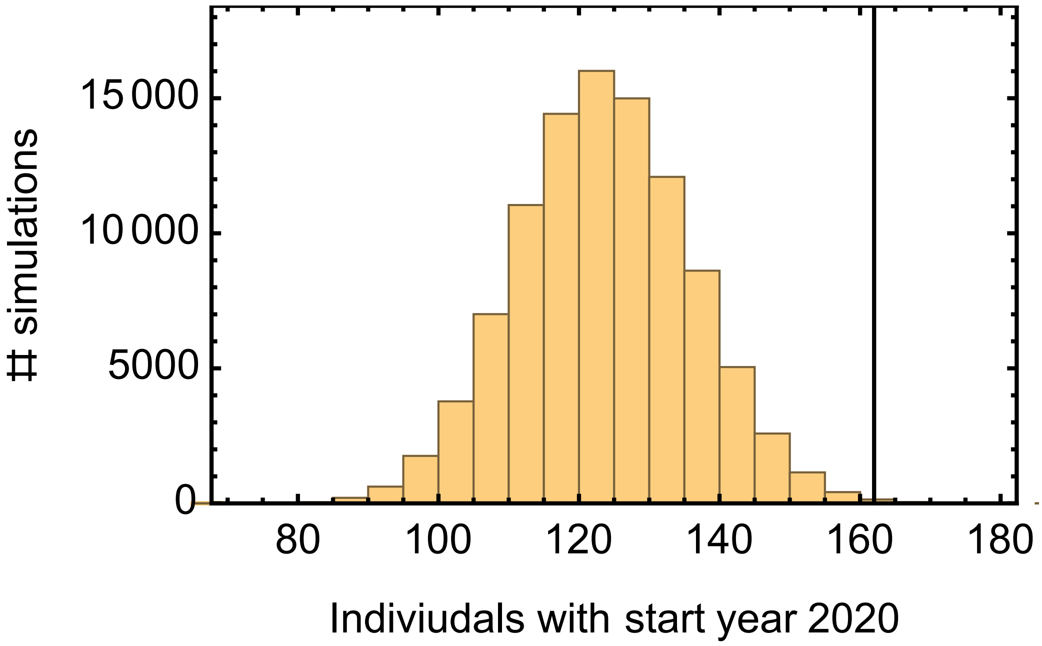

Supplementary Figure 13: Simulation of number of 1st year cases expected to be seen by random chance in the large University Cluster (cluster 1). The data shows an over-representation of individuals with start year 2020 in the data (vertical black line) compared to the neutral expectation (histogram) (one-tailed test, p-value 0.0.002). Simulations were used to evaluate the significance or otherwise of this bias. To account for household structure, we examined the year groups of infected students who shared accommodation. From 261 cases in which more than one individual in a household was infected, we calculated that there was a 90% probability that two pairwise individuals in a given household were from the same year group. We next simulated outbreaks across the households in our study, using the numbers of infections identified in each house. For the house i, we randomly assigned the first case to be from a student in their first, second, or later year according to the proportions of students in each year group (33.36%, 26.73%, and 39.91% respectively). For the same house, we then assigned each other case to be from the same year as the first case with 90% probability, assigning the year of the student in proportion to the numbers of students in other years if not. This process was repeated 10^5 times, giving a distribution of outcomes measured in terms of the number of students in their first year of study produced by the simulated outbreak, giving the distribution shown in Supplementary Figure 13. Of these simulated outbreaks, 99.8% had fewer first year students infected than did our real dataset. Source data are provided as a Source Data file.

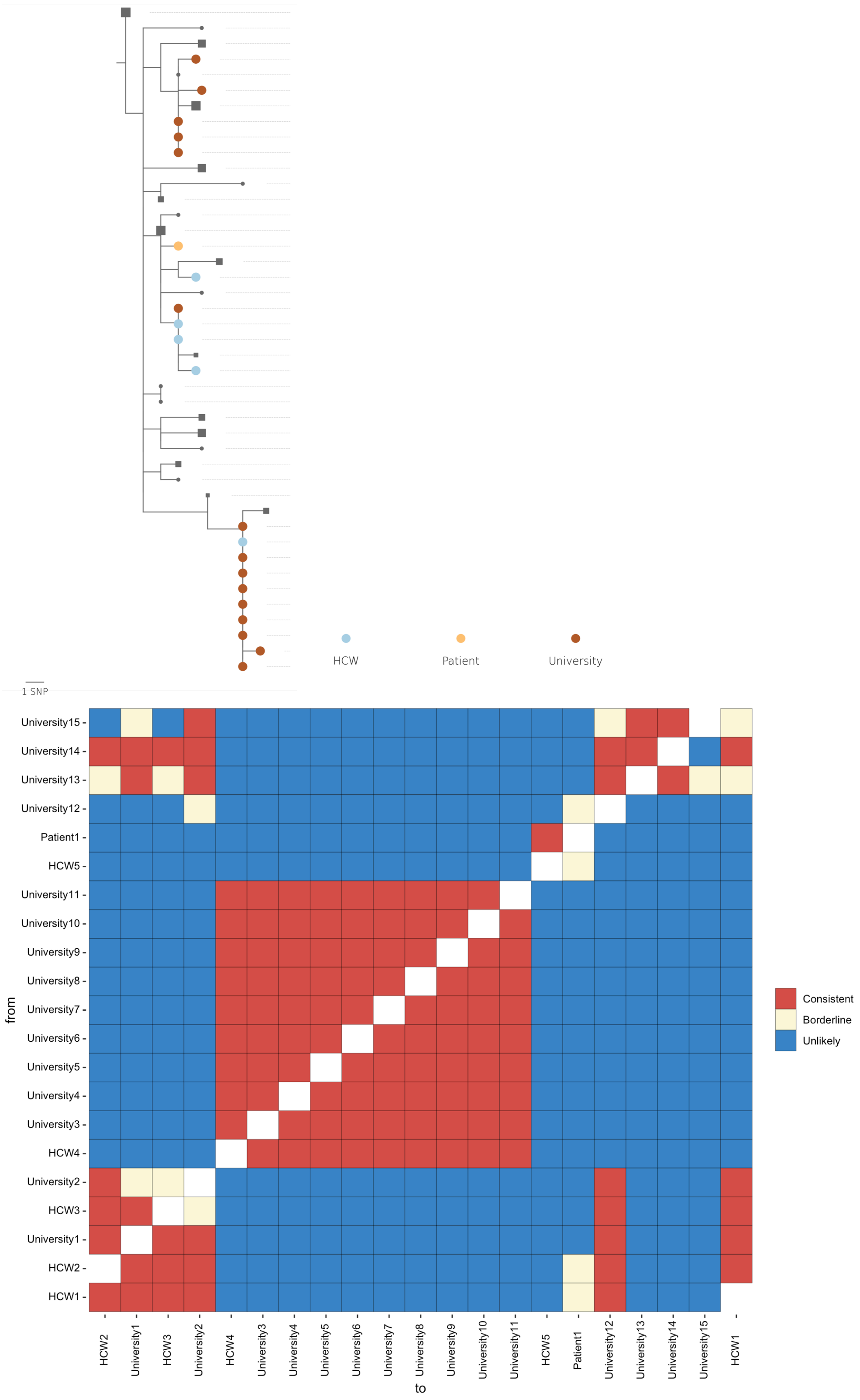

Supplementary Figure 14: CIVET phylogenetic tree of a Cambridge Universities Hospital (CUH) cluster amongst healthcare workers (HCW) and medical students, with associated A2B-COVID output. Both demonstrate a large cluster of individuals linked to the hospital setting, with consistent transmission seen between multiple medical students and a HCW and separately between a group of patients and HCWs. Sequences from GISAID used to

contextualise study sequences by CIVET are represented by dark grey squares (collapsed nodes) and dark grey circles (individual sequences). HCW = Healthcare worker.

Supplementary Tables for University Genomics Paper

Supplementary Table 1a. Summary of the 16 genomic clusters of two or more university cases, generated using the clustering tool CIVET (UoC = University of Cambridge; HCW = healthcare workers).

Cluster	Lineage	Number of cases					Epiweek in which cases identified						Notes
							Whole cohort		UoC		Community		
		Total	UoC	HCW	Patients	Community	First Case	Last Case	First Case	Last Case	First Case	Last Case	
1	B.1.160.7	354	337	1	1	15	41	49	41	49	43	48	See supplementary table 1b below
2	B.1.36	32	30	0	0	2	41	43	42	43	41	41	Of the 30 university members: 24 were students living in the same accommodation block in College A; 4 were students living in different accommodation in the same College, 3 of whom were living in the same household; 2 students in different colleges had no identified associations with other cases in this cluster.
3	B.1.177.16	35	20	0	0	15	41	50	42	47	41	50	Of the 20 university members: 5 belong to 2 households on neighbouring staircases in College B; 4 belong to 2 neighbouring households in a different accommodation block in College B; 1 further student is resident in a different accommodation block in College B; 2 further cases from College C, and one from College D share the same course and year of study as a student from College B; the 2 students in College C are named contacts of each other in university contact tracing and share a common exposure with an individual at College B in national contact tracing; a student from College E lives in the same household as a student whose isolate did not sequence, but who is on the same course as the students from Colleges C and D and is named in university contact tracing by the student in College D; 2 further students from College A live in the same household but, as with the remaining two students, have no identified epidemiological associations with any other student in this cluster. No further growth of the cluster is seen amongst students after week 3, but 2 infections are noted in week 7, both in university staff members who share a household.
4	B.1.177	201	25	38	30	108	40	51	41	49	40	51	Of the 25 university members: 2 share the same household in College G; 1 student shares the same course and year of study as one of the students from College G; 2 students in separate colleges are in the same year and course as each

													other; 2 staff members work in the same college (no students are identified from this college) and live very close to one another; 4 students are clinical medical students in the same block of accommodation in College H; a 5th clinical medical student is from College H but lives in a different household and is in a different year of study; 7 are clinical medical students in neighbouring households in College I; 2 further clinical students in different colleges are named contacts of the index case in College I; 2 students and 2 staff members have no obvious association with anyone else in this cluster
5	B.1.177	7	4	0	0	3	44	48	44	45	45	48	All 4 of the university members are students living in the same household
6	B.1.177	4	3	0	0	1	46	46	46	46	46	46	Of the 3 university members: 2 are students in the same college, but different households and courses; there is no evident association with the 3rd case, a member of staff
7	B.1.177	4	2	0	0	2	43	45	45	45	43	45	The 2 university members are students who share the same household and course
8	B.1.177	3	3	0	0	0	45	45	45	45			Of the 3 university members: 2 are students in the same household and course; there is no evident association with the 3rd student
9	B.1.177.17	41	6	2	2	31	40	50	43	48	40	50	Of the 6 university members: 2 are members of staff in the same college; 1 of these lives in the same household as another individual in this cluster; there is no evident association with the other 3 members
10	B.1.177	10	9	0	0	1	42	45	42	44	45	45	Of the 9 university members: 2 share the same course and year of study in College J; 1 further student in College J shares the same course, but is in a different year of study; 3 further students in College K share the same course as the 2 students in College J, with 2 being in the same year of study; 1 further student in College J shares the same year of study with other students, but is on a different course; 1 further student in College K shares the same year of study, but a different course; one student in College C is a named contact of a student from College J; 1 student has no identified association with any other students.
11	B.1.177.4	19	7	0	1	11	44	47	45	47	44	47	Of the 7 university members, 4 are staff and 3 are students; 2 staff work in the same 'additional personnel' department. There is no identified association between the remaining members of this cluster
12	B.1.1.315	21	6	3	1	11	39	47	45	47	39	46	Of the 6 university members: 2 students share the same household and are both PhD students in the same department; a 3rd student is also a PhD student in this department but living in a different household and college; 2

													individuals share the same household, but like the remaining individual have no association with the other cases in this cluster
13	B.1.258	12	3	0	0	9	42	46	42	42	44	46	Of the 3 university members: 2 students share the same postgraduate course and work in the same department; there is no identified association with the third student
14	B.1	5	3	0	0	2	45	48	46	46	45	48	Of the 3 university members: 2 staff members share the same household; there is no identified association with the third member, a student
15	B.1.177	27	2	2	1	22	41	49	47	48	41	49	Both university cases are staff members with no identified associations
16	B.1.1.153	4	2	0	0	2	41	44	42	42	41	44	Both university cases are students in the same academic year with no other identified associations

Supplementary Table 1b. Summary of the 19 genomic clusters of two or more university cases, using a SNP difference threshold of 0 SNPs, based on isolates from cluster 1 identified by CIVET in table A above (UoC = University of Cambridge; HCW = healthcare workers).

Cluster	Lineage	Number of cases					Epiweek in which cases identified						Notes
							Whole cohort		UoC		Community		
		Total	UoC	HCW	Patients	Community	First Case	Last Case	First Case	Last Case	First Case	Last Case	
A	B.1.160.7	182	176	0	0	6	44	48	44	47	46	48	There are a large number of cases that emerge in the same week, making further analysis challenging. Of the 176 university members: 113 are students sharing a household with at least one other individual in this cluster; the largest household cluster is 11 students living on the same staircase; 155 students share a course and year of study with at least one other individual in the cluster, with some overlap with college household structure; the largest cluster sharing course, year of study and college is 7 students.
B	B.1.160.7	6	6	0	0	0	46	48	46	48			Of the 6 university members: all 6 are students living in shared or neighbouring households in the same college; 3 of these students are in the same course and year of study
C	B.1.160.7	2	2	0	0	0	46	47	46	47			The 2 university members are students that share the same year and college, and are identified contacts in university contact tracing
D	B.1.160.7	2	2	0	0	0	46	46	46	46			Both university cases are students, but have no identified association.

E	B.1.160.7	62	60	0	1	1	41	46	41	46	44	46	Of the 62 university members: 21 students share a household with at least one other individual in this cluster; the largest household cluster is 4 students living on the same staircase; 29 students share a course and year of study with at least one other individual in the cluster, with some overlap with household structure; the largest cluster sharing course and year of study is 5 students; of note, two students in the first week that this cluster was identified report attending Venue A on the same day in the first week of term.
F	B.1.160.7	3	3	0	0	0	44	45	44	45			Of the 3 university members: 2 students share the course and year of study; there is no identified association with the 3rd student.
G	B.1.160.7	2	2	0	0	0	42	42	42	42			Both university cases are students, but have no identified association.
H	B.1.160.7	3	3	0	0	0	46	46	46	46			All 3 university cases are students, but have no identified association.
I	B.1.160.7	2	2	0	0	0	46	46	46	46			Both university cases are students, and share the same household and course
J	B.1.160.7	4	4	0	0	0	44	45	44	45			Of the 4 university members: 3 are students from the same college (2 in the same household) but different courses; 1 member of staff has no known associations with the students
K	B.1.160.7	2	2	0	0	0	44	44	44	44			Both university cases are students, and share the same course and year of study
L	B.1.160.7	2	2	0	0	0	43	44	43	44			Both university cases are students, but have no identified association.
M	B.1.160.7	2	2	0	0	0	44	44	44	44			Both university cases are students, but have no identified association.
P	B.1.160.7	9	9	0	0	0	45	48	45	48			Of the 9 university members: 4 live in the same/neighbouring households in College L; 2 live in neighbouring households from College J; a further student is also at College J; 2 the remaining 2 students have no identified association with the rest of the cluster
Q	B.1.160.7	13	12	0	0	1	44	46	44	46	46	46	Of the 13 university members: 3 are students on the same course and year of study (1 is named as a contact of the first; another is in the same college as the first); 6 live in the same block of accommodation in a different college; the 4 remaining members have no identified association.
R	B.1.160.7	4	4	0	0	0	45	47	45	47			Of the 4 university members: 2 students live in the same household; a 3rd students lives in the same college on a different course and year of study; 1 of the students has no identified association with other members of this cluster; one of the students lives in the same household and shares a

													course and year of study with a student in cluster Q, which is one SNP different from R
S	B.1.160.7	18	15	0	0	3	43	45	43	45	43	45	Of the 15 university members: 5 are students sharing the same course and household at College L; 2 live in a different household at College L; 4 live in the same accommodation block in College M; 4 live in College N, of which 1 is a named contact of another in university contact tracing; 1 of the students in College L and another at College M share the same course and year of study
T	B.1.160.7	2	2	0	0	0	41	41	41	41			These are the earliest 2 isolates from this large cluster during the study. There is no identified association between these 2 students; 1 student has an isolate that is 1 SNP different from a household contact from cluster E

Supplementary Methods and Results

Study Setting

Colleges
All students live, eat and socialise in one of the university's 31 autonomous colleges. Undergraduates receive supervisions (small group teaching sessions) in their colleges. Most colleges admit both undergraduate and postgraduate students[3].

Households
In this study, households are defined as individuals who share a kitchen, bathroom and/or lounge facilities, in line with national and UoC guidance distributed to all colleges.

Course structure
Course groupings are defined as[4]:

- Undergraduate arts and humanities – undergraduate students in the School of Arts and Humanities and the School of Humanities and Social Sciences
- Undergraduate science and technology – undergraduate students in the School of Biological Sciences, the School of Physical Sciences and the School of Technology
- Postgraduate vocational courses – students in clinical medicine, clinical veterinary medicine and postgraduate certificates in education
- Other postgraduate courses – all other postgraduate students, including those in doctoral and masters programmes.

Year group structure
This is described in more detail elsewhere[5].

Community
The University of Cambridge is situated in the city of Cambridge, Cambridgeshire. Cambridgeshire has a total population estimate for 2019 of 855,796, with approximately 428,132 (50%) males[6]. 90.3% of the population identified as White in the last population census[7].

University participants and samples

Isolates for this study were derived from the symptomatic testing programme and asymptomatic COVID-19 screening programmes within the UoC between 5 October 2020 and 6 December 2020, covering the full term. Testing for all symptomatic students and staff within the university has been available on all weekdays from 5th October. The asymptomatic screening programme has been described in detail elsewhere[5]. In brief, during the study period screening was offered on a voluntary basis to all students resident in accommodation owned or managed by a College or the Cambridge Theological Federation. In total, 15,561 students were eligible to participate. To optimise efficiency of testing, swabs were pooled into the same tube of viral transport medium at the time of sample collection. Testing pools vary in size from 1 to 10 students, based on student households[5]. The individual members of any positive pool were re-tested using individual confirmatory PCR tests. Only positive samples from the individual confirmatory tests were taken forward for sequencing.

All SARS-CoV-2 tests for UoC students were performed by PCR in established UoC/AstraZeneca Cambridge COVID-19 Testing Centre in the Anne McLaren Building, Cambridge Biomedical Campus, part of the UK Lighthouse Labs Network, using the same procedures as those used in national community testing. All plates containing extracted RNA from university samples were shipped to the UoC Department of Medicine, so that positive

samples with a Ct value ≤33 were picked and sequenced using the GridION platform (Oxford Nanopore)[8] (see below).

Throughout the study period, confirmed individual cases were notified to NHS Test and Trace and the UoC COVID helpdesk for parallel contact tracing efforts. On the day of their test result, all confirmed cases were asked to complete a monitoring form held centrally by the university, which included documentation of contacts who were at risk of transmission. Contacts were reached by the university or their college to inform them of the need for immediate isolation. In all cases, isolation of cases and contacts was in accordance with UK national guidance. In brief, household members and other high-risk contacts of confirmed cases quarantined for 14 days, while the cases themselves self-isolated for 10 days from date of symptom onset, or date of test, if asymptomatic. Colleges provided support for student isolation; the nature of this support varied between colleges and households, but included provisions such as food and drink, educational and psychological support where required.

In addition to offering symptomatic testing and asymptomatic screening, UoC supported a number of COVID-19 reduction measures for the duration of the study period under the banner of their “StaySafeCambridgeUni” campaign. These measures reflected national UK policy at the time and were communicated to students prior to the start of term, periodically reinforced as part of routine email communications from the University and Colleges or Departments during the term and supported by posters across the University campus. All University policies and a range of supporting materials were made available online. Additional support and information was provided by Colleges, University departments and through a network of collegiate student representatives and the university-wide student union.

COVID-19 reduction measures included:

- The mandatory use of face coverings in work or study settings, indoor meetings and teaching sessions lasting longer than 15 minutes, unless the individual concerned had a medical exemption;
- Widespread promotion of hand washing and availability of virucidal hand sanitisers;
- Guidance on social distancing for all individuals apart from household contacts, to a minimum of 2 metres;
- Moving lectures and other large group teaching online. Small group teaching and practical classes were continued in accordance with the policies above.

All international students were asked to self-isolate immediately on arriving in Cambridge for a period of 14 days.

From the start of the study period until November 4, extra-curricular, sporting and social activities were permitted so long as the above guidance could be followed. There were no additional restrictions placed on student movement between the University and the local community. Individual Colleges made policies on the admission of the general public and members of other Colleges into their premises.

Overall support and coordination for the University's COVID-19 response was provided by a dedicated helpdesk, providing telephone and e-mail advice and decision-making for students and staff. Oversight was provided by senior members of the University in a gold-silver-bronze command structure.

Representatives of the University and the COVID-19 helpdesk met with members of the local public health authorities at least twice-weekly during the study period to monitor surveillance activities and coordinate interventions to reduce transmission. Where high rates of transmission were identified in a particular college, additional meetings were held with representatives from all parties. Numbers of newly confirmed cases where shared daily between the screening programme, symptomatic testing, COVID-helpdesk, public health authorities and members of the University's gold command.

Specific measures to reduce transmission following the identification of an outbreak (defined as two or more epidemiologically linked cases) were tailored to each individual circumstance, but followed similar principles. For example, one of the first clusters of cases to be identified within a single block of accommodation in one college and managed as an outbreak was detected during the second week of term. The first individuals to be identified were screened via the asymptomatic screening pathway. Over the following 2 days further students were identified through the university's symptomatic testing route. All suspected and confirmed cases, and their households, were immediately isolated and contact tracing initiated as described above. Subsequent genomic analysis confirmed that the majority of these cases were linked isolates, and are described in the results as cluster two. Within four days of the index case testing positive an extraordinary meeting was held between members of the college, university and the local public health authority who agreed an immediate lockdown of the affected accommodation block in its entirety. Students were supported with deliveries of food and drink, their educational needs were discussed individually between students and their tutors, and additional psychological support was provided as necessary. However, students were not allowed to leave the accommodation block unless they were attending an appointment for SARS-CoV-2 testing or another valid medical reason. In addition to the existing availability of symptomatic testing from the university, individual asymptomatic screening was offered to all students living in the accommodation block over the following four days. These measures were successful at reducing the number of cases within both the accommodation block and the wider college. As described in Results, subsequent genomic analysis has demonstrated this viral lineage became extinct in the study population within two weeks of the accommodation block being placed under isolation.

Announced on October 31 2020, a national lockdown was declared by the UK government on November 5 which lasted until December 1. Stricter restrictions were put in place during this time, including the closure of all hospitality venues, limitations on mixing between households (unless students were part of an existing social bubble) and movements outside the home unless for essential activities (such as shopping or medical care) or physical exercise. During this time all sporting activities were cancelled, as were social activities involving multiple households. The majority of in-person teaching was either postponed or moved online, with the exception of students on vocational training programmes such as clinical medical students. Further pastoral support was provided through the colleges.

Cambridge University Hospital sample selection

CUH samples underwent one of two testing methods as they became available during the study. In method one samples underwent nucleic acid extraction and were tested for presence of SARS-CoV-2 using a validated in-house RT qPCR assay developed by Public Health England Clinical Microbiology and Public Health Laboratory (CMPHL)[9]. The test was reported as SARS-CoV-2 PCR positive if the cycle threshold (Ct) value was ≤36. Method two utilised an automated, proprietary PCR based assay (Hologic, Panther) validated to the

CMPHL in-house RT qPCR assay. SARS-CoV-2 positive samples were considered to be any sample with an RLU value ≥600.
All PCR-positive diagnostic samples were identified and transferred from the CMPHL to the Division of Virology for nanopore sequencing. All CUH samples were selected for sequencing. Samples from methods two and three underwent additional RNA extractions to isolate viral RNA from proprietary solutions.

Sample preparation for sequencing of University of Cambridge and CUH samples

Samples identified were sequenced using a multiplex PCR-based approach according to the modified ARTIC v2 protocol and with either the v2 or v3 primer set as they became available[10]. Sample preparation, barcoding, adapter ligation and clean up were completed according to the modified ARTIC v2 protocol as it was developed[10]. As a correlation between amplicon concentration and genome coverage was established, an additional quality control step was created to screen out samples with amplicon concentrations lower than 5 ng/µL.

Sequencing and assembly of University of Cambridge and CUH samples

Amplicon libraries were sequenced using MinION flow cells v9.4.1 (Oxford Nanopore Technologies, Oxford, UK). Genomes were assembled using reference-based assembly to the *MN908947.3* sequence and the ARTIC bioinformatic pipeline using 20x minimum coverage cut-off for any region of the genome and 50.1% cut-off for calling single nucleotide polymorphisms (Loman *et al.* 2020; Meredith *et al.* 2020; Wu *et al.* 2020).

Metadata association and quality control of University of Cambridge and CUH samples

Assembled genomes were associated to demographic, clinical, and laboratory data by CMPHL and uploaded to the Medical Research Council (MRC) Cloud Infrastructure for Microbial Bioinformatics Samples (CLIMB) database[11]. Samples with ≥70% genome coverage and associated metadata were accepted by the COG-UK. Samples with ≥90% genome coverage and associated metadata were further uploaded to the Global Initiative on Sharing All Influenza Data (GISAID)[12]. Samples with ≥95% genome coverage were included in the study for phylogenetic analysis.

Additional sequences derived for from pooled testing term week 1

Individual samples from students identified as being SARS-CoV-2 positive through asymptomatic screening were not available for the first week of term (week commencing 5th October). Given the potential importance of identifying lineages present in the university in the first week of term, attempts were made to sequence all RNA extracts from the pooled samples where an individual positive student had been identified. This yielded an additional 6 sequences derived from pooled samples, of which 5 samples were associated with 1 individual positive student on confirmatory testing. One pooled sample was associated with two individual positive students, with individual CT values of 21.6 and 21.9.

List of Definitions

Cluster = A cluster was defined with default CIVET settings, extracting phylogenetic neighbours to represent a possible chain of transmission between isolates (within 2 nodes of one another). Further details are found on https://github.com/artic-network/civet.
Sub-cluster = Isolates with '0' SNP differences within a transmission cluster. Given the size of Cluster 1 and its transmission across the entire university term, the cluster was further evaluated to provide additional context to epidemiological data by grouping individuals with 0 SNP differences between SARS-CoV-2 isolates.
Lineage = Global Pango Lineages[13] were assigned to each genome using Pangolin v2.1.6 (https://github.com/cov-lineages/pangolin) with analyses performed on COVID-CLIMB[11]. Pango lineages are denoted with a letter followed by a hierarchy of up to 3 numbers, such as B.1.2.3, providing for a stable and consistent naming of clusters. These lineages are manually curated and assigned.

Common Exposure = Locations or activities reported by two or more cases in the 2 to 7 day period before symptom onset or test date if symptom onset date is not provided. Events are matched based on activity/setting post code and event category. Data is gathered on household, workplace, education and recreational activities. Individuals are grouped into a common exposure when they matched to a location within a 7-day rolling period. Data is gathered by test and trace. Data was manually reviewed to ensure accuracy.

Household = A university household included individuals in college accommodation with a shared bathroom, kitchen, or lounge facility.

Birth-death skyline model robustness

To evaluate the robustness of the R_e and effective infectious period estimates of cluster 1 we used different parameterisations of the birth-death skyline model. The sampling proportion was (i) fixed to the empirical estimates (number of sequenced genomes from all University clusters divided by the number of positive tests among University staff and students), (ii) fixed to 0 before the start of term and estimated for each week thereafter, and (iii) fixed to 0 before the first week of term and assumed to be constant thereafter (**Supplementary Figure 8**). Next, R_e was (i) parameterised into 20 epochs, equidistantly spaced between the origin time and the most recent sequence collection date, and (ii) assumed to be constant before the first week of term and estimated for each week thereafter (**Supplementary Figure 8 and 9**). Finally, different sampling proportion priors were used, (i) Beta(2.5, 5), (ii) Beta(5,5) and (iii) Beta(7.5, 2.5) (**Supplementary Figure 11**).

Cluster 1 Year Group Analysis

We used a simulation-based method to evaluate whether the year in which a student began their studies was statistically related to the probability of their testing positive for COVID infection. A substantial proportion of transmission events take place within a single household, and individuals of the same year group are potentially more likely to share a household. For this reason, we first assessed the relationship between starting year and sharing accommodation.

From the list of students who were detected as being infected with COVID, we identified households in which more than one individual was infected, and for which information describing the start year of each student was available; this identified a total of 81 households. Across these households, 261 pairs of individuals within the same house existed, of which 234 were in the same year group. This gave an estimated 90% probability that a pair of individuals in the same house were in the same year group.

We next used a simulation method to evaluate the distributions of year groups of individuals within clade 160. The starting years of individuals with viruses in clade 160 were identified. These were predominantly undergraduate students within their first three years of study. Starting years were distributed as follows:

2020	162
2019	85
2018	74
2017 or earlier	8
Not available	6

To evaluate the significance of the predominance of individuals who began their studies in 2020, we compared the results above to those generated from a neutral model, in which students starting between 2018 and 2020 had a probability of being infected equal to the fraction of individuals of that year who participated in the study; the study included 3336 individuals with start year 2020, 2673 with start year 2019, and 2434 with start year 2018.

The household structures of students with start dates between 2018 and 2020 were identified, being classified as the number of infected students per household.

Infections	1	2	3	4	5	6	7
Households	146	34	8	4	8	2	1

We conducted a repeated random sampling of the outbreak within this household structure. In each simulation we drew the start year of the first student in each household according to the fraction of individuals in that year who participated in the study. The remaining cases in each household were then sampled, with a 90% probability of being of the same starting year as the first case, and chance of being in either of the two starting years calculated accordingly, and in proportion with the number of participating individuals of that year in the study.

Running this sampling process 100,000 times provided an empirical distribution of the number of individuals in each year group expected under a neutral model, given the underlying household structure of the observed outbreak. The data shows an over-representation of individuals with start year 2020 in the data (line) compared to the neutral expectation (**Supplementary Figure 13**) (p-value 0.002).

Household Secondary Attack Rates

We combined genome sequencing and epidemiological data to estimate a within-household force of infection. Firstly, the A2B-COVID package was used to identify households for which the sequence and timing information was consistent with a single introduction of the virus to the household, or when no sequence data was available, with timing data alone. Secondly, collecting data from each such household, a chain binomial model was used to estimate the probability that an infected person passed on the virus to an uninfected person within the same household.

A2B-COVID

The A2B-COVID package uses data from multiple sources to evaluate whether data from two individuals is consistent with direct transmission having occurred between those two individuals. Given two individuals A and B, we denote the data by y, and X as the event that transmission took place from A to B. We then calculate a conditional probability P(y|X), comparing this value to thresholds $P_{0.95}$ and $P_{0.99}$, which denote 95% and 99% thresholds for rejecting the hypothesis that transmission from A to B occurred. From the data y we thus infer an estimate of whether the data are consistent with transmission from A to B ($P(y|X) < P_{0.95}$), whether the data are unlikely to have been observed from a transmission event ($P(y|X) \geq P_{0.99}$), or whether an event is borderline ($P_{0.95} \leq P(y|X) < P_{0.99}$).

For a given pair of individuals, we have that:

$$p(y|D,X) = \sum_T \quad P(T|\widehat{S_A},\theta)P(\widehat{S_B}|\theta,X_T)P(H_A,H_B|\,\theta,D,X_T),$$

where T denotes the time of transmission and X_T is the event that transmission occurred on day T, $\hat{S}_A$ is the estimated day on which individual A became symptomatic, H_A is the Hamming distance from the viral sequence collected from individual A to the consensus of

the sequences from A and B, D={D_A,D_B} describes the times at which viral sequences were collected from A and B, and θ is used as shorthand for a series of other parameters described below.

$$P(T|S_A,\theta)P(S_B|\theta,X_T) = \left[\int_{T-S_A-0.5}^{T-S_A+0.5} \frac{e^{-(x+s)/\beta}(x+s)^{\alpha-1}\beta^{-\alpha}}{\Gamma(\alpha)}dx\right]\left[\int_{S_B-T-0.5}^{S_B-T+0.5} \frac{e^{-\frac{(log(x)-\mu)^2}{2\sigma^2}}}{x\sigma\sqrt{2\pi}}dx\right].$$

Here, we used parameters derived from previous literature: α=97.1875, β=0.2689, s=25.625, μ=1.434, and σ=0.6612[14-16].

while

$$P(H_A,H_B \mid \theta,D,E,X_T) = \left(\frac{(E/2+\gamma_G P_A)^{H_A} e^{-(E/2+\gamma_G P_A)}}{H_A!}\right)\left(\frac{\left(E/2+\gamma_G(D_B-Q_A)\right)^{H_B} e^{-(E/2+\gamma_G(D_B-Q_A))}}{H_B!}\right)$$

where E/2 is the estimated number of nucleotide errors in a viral genome sequence collected using the ARCTIC nanopore sequencing protocol, estimated in a previous publication as 0.414[17], γ_G describes the expected number of substitutions per genome per day, calculated as 0.0655 using an estimate of the global rate of viral evolution[18] , P_A = max{0, D_A - T} and Q_A = min{D_A, T}.

Chain binomial model

Households of infected individuals in our study ranged in size from 1 to 18. We first define the terms H_N, the number of households of size N>1, and H_{Nj}, the number of households of size N>1 for which j individuals were infected.

We next applied a simple Reed-Frost chain binomial model, presupposing multiple rounds of infection within the household. We denote by p the probability that an infected individual infects a previously uninfected individual in the same household.

If in round i of this process a total of n_i individuals were infected, we have that there remain

$$M_i = N - \sum_{a=1}^{i} n_i$$

individuals to be infected. Further, the probability of one of those individuals being infected by an individual infected in round i is given by

$$q_i = 1-(1-p)^{n_i}$$

We may therefore say that n_{i+1} individuals are infected in round i+1 with probability

$$\frac{M_{i!}}{n_{i+1}!\,(M_{i-}n_{i+1})!} \quad q_i^{n_{i+1}}\left(1-q_i\right)^{n_{i+1}}$$

The above process terminates as soon as no individuals are infected in round i. Using the above formulation, we denote the total number of individuals infected in a household, $n = \sum_i \quad n_i$,
and calculate the conditional probabilities

$$P_{N,p}^{k} = P(n=k|N,p)$$

The likelihood of the value p is then given by the sum of multinomial likelihoods

$$log\ L = \sum_{N=2}^{18} \ log \left[\frac{H_N!}{H_{N1}!\, H_{N2}! \ldots H_{NN}!} \prod_{j=1}^{N} \ \left(P_{N,p}^{j}\right)^{H_{Nj}} \right]$$

A simple optimisation method was used to maximise this likelihood.

Supplementary References

1. Seemann, T., *et al.* Tracking the COVID-19 pandemic in Australia using genomics. *Nat Commun* **11**, 4376 (2020).
2. Duchene, S., *et al.* Temporal signal and the phylodynamic threshold of SARS-CoV-2. *Virus Evol* **6**, veaa061 (2020).
3. Cambridge, U.o. About the University: Structure. Vol. 2021 (2021).
4. Cambridge, U.o. Colleges and Departments. Vol. 2021 (2021).
5. Warne, B. Feasibility and efficacy of mass testing for SARS-CoV-2 in a UK university using swab pooling and PCR testing. *bioRxiv* (2021).
6. Statistics, O.f.N. Cambridgeshire and Peterborough Population Overview Report. Vol. 2021.
7. Statistics, O.f.N. 2011 ONS Census. Vol. 2021.
8. Tyson, J.R., *et al.* Improvements to the ARTIC multiplex PCR method for SARS-CoV-2 genome sequencing using nanopore. *bioRxiv* (2020).
9. Hamilton, W.L., *et al.* Genomic epidemiology of COVID-19 in care homes in the east of England. *Elife* **10**(2021).
10. Quick, J. nCoV-2019 sequencing protocol v3 (LoCost) V.3. Vol. 2021 (2020).
11. Connor, T.R., *et al.* CLIMB (the Cloud Infrastructure for Microbial Bioinformatics): an online resource for the medical microbiology community. *Microb Genom* **2**, e000086 (2016).
12. Shu, Y. & McCauley, J. GISAID: Global initiative on sharing all influenza data - from vision to reality. *Euro Surveill* **22**(2017).
13. Rambaut, A., *et al.* A dynamic nomenclature proposal for SARS-CoV-2 lineages to assist genomic epidemiology. *Nat Microbiol* **5**, 1403-1407 (2020).
14. Li, Q., *et al.* Early Transmission Dynamics in Wuhan, China, of Novel Coronavirus-Infected Pneumonia. *N Engl J Med* **382**, 1199-1207 (2020).
15. He, X., *et al.* Author Correction: Temporal dynamics in viral shedding and transmissibility of COVID-19. *Nat Med* **26**, 1491-1493 (2020).
16. Ashcroft, P., *et al.* COVID-19 infectivity profile correction. *Swiss Med Wkly* **150**, w20336 (2020).
17. Illingworth, C.J.R., *et al.* A2B-COVID: A method for evaluating potential SARS-CoV-2 transmission events. *medRxiv* (2020).
18. Hadfield, J., *et al.* Nextstrain: real-time tracking of pathogen evolution. *Bioinformatics* **34**, 4121-4123 (2018).
