# Supplementary material for: Genomic epidemiology of SARS-CoV-2 in a UK university identifies dynamics of transmission
Source: Nat Commun. 2022 Feb 8;13:751. doi: 10.1038/s41467-021-27942-w (PMC8826310; doi:10.1038/s41467-021-27942-w)
Supplement: Supplementary file 3 — Description of Additional Supplementary Files [file 41467_2021_27942_MOESM3_ESM.pdf]

Description of Additional Supplementary Files

File Name: Supplementary Data 1
Description: ENA accession codes of sequences used in this study

File Name: Supplementary Data 2
Description: Genbank accession codes of pooled sample sequences used in this study
