# Supplementary material for: Genomic epidemiology of SARS-CoV-2 in a UK university identifies dynamics of transmission
Source: Nat Commun. 2022 Feb 8;13:751. doi: 10.1038/s41467-021-27942-w (PMC8826310; doi:10.1038/s41467-021-27942-w)
Supplement: Supplementary file 6 — Reporting Summary [file 41467_2021_27942_MOESM6_ESM.pdf]

nature research

Corresponding author(s): Dinesh Aggarwal, Ewan Harrison, Nick J Matheson, Ian G Goodfellow

Last updated by author(s): Nov 12, 2021

Reporting Summary

Nature Research wishes to improve the reproducibility of the work that we publish. This form provides structure for consistency and transparency in reporting. For further information on Nature Research policies, see our Editorial Policies and the Editorial Policy Checklist.

Statistics

For all statistical analyses, confirm that the following items are present in the figure legend, table legend, main text, or Methods section.

n/a	Confirmed	
☐	☒	The exact sample size (*n*) for each experimental group/condition, given as a discrete number and unit of measurement
☒	☐	A statement on whether measurements were taken from distinct samples or whether the same sample was measured repeatedly
☐	☒	The statistical test(s) used AND whether they are one- or two-sided<br>*Only common tests should be described solely by name; describe more complex techniques in the Methods section.*
☒	☐	A description of all covariates tested
☒	☐	A description of any assumptions or corrections, such as tests of normality and adjustment for multiple comparisons
☐	☒	A full description of the statistical parameters including central tendency (e.g. means) or other basic estimates (e.g. regression coefficient) AND variation (e.g. standard deviation) or associated estimates of uncertainty (e.g. confidence intervals)
☐	☒	For null hypothesis testing, the test statistic (e.g. *F*, *t*, *r*) with confidence intervals, effect sizes, degrees of freedom and *P* value noted<br>*Give P values as exact values whenever suitable.*
☐	☒	For Bayesian analysis, information on the choice of priors and Markov chain Monte Carlo settings
☒	☐	For hierarchical and complex designs, identification of the appropriate level for tests and full reporting of outcomes
☒	☐	Estimates of effect sizes (e.g. Cohen's *d*, Pearson's *r*), indicating how they were calculated

Our web collection on statistics for biologists contains articles on many of the points above.

Software and code

Policy information about availability of computer code

Data collection	Where indicated, collapsed nodes from trees generated from CIVET (version 2.1.0) were inspected to visualise data in the context of the COG-UK national database (https://www.cogconsortium.uk/).

Data analysis

Samples were prepared using ARTIC or veSeq protocols, and were sequenced using Illumina or Oxford Nanopore platforms. Samples derived from University participants were sequenced on a Nanopore GridION Mk1 running Minknow 20.10.6 (Oxford Nanopore Technologies, Oxford, UK) and real time read mapping was visualised with RAMPART v1.2.0 (https://artic-network.github.io/rampart/). Sequences were assembled using the ARTIC pipeline (Nanopolish) v1.0.0 (https://github.com/artic-network/fieldbioinformatics). Genomes were aligned with minimap2. All samples were processed through COVID-CLIMB pipelines. Protocols are available at https://github.com/COG-UK. Maximum likelihood phylogenetic trees were estimated using IQ-TREE (version 2.1.2 COVID-edition). TempEst (v.1.5.3) was used to explore the temporal signal in the data. Trees were visualised, explored, and labelled with associated metadata using Microreact (https://microreact.org/). Specified mutations were identified using type_variants (https://github.com/cov-ert/type_variants). Clusters were defined using the CIVET tool (version 2.1.0) on 2021-01-11 (https://github.com/artic-network/civet). In selected clusters, further evaluation was conducted using A2B-COVID (v0.1.0). Pairwise SNP differences between sequences were determined using SNP-dist (https://github.com/tseemann/snp-dists/releases/tag/v0.7.0). Global Pango Lineages were assigned to each genome using Pangolin (https://github.com/cov-lineages/pangolin/releases/tag/v2.1.6) with analyses performed on COVID-CLIMB. BEAST v1.10.4 was used to perform a time-scaled phylogenetic analysis, convergence was assessed using Tracer. Maximum clade credibility (MCC) tree was generated using TreeAnnotator (v1.10.4). A Bayesian birth-death skyline (BDSKY) model was employed for analysis using BEAST v2.6, convergence was assessed using the R-package coda. Epidemiological data from the University of Cambridge were initially compiled in Microsoft Azure SQL and Excel 2013 (Microsoft) and analysed in STATA 14.2 (College Station, TX, USA). Further data manipulation, statistical analysis and figure generation was undertaken with RStudio (version 1.3.1093) using R (version 4.0.2). Network diagrams were produced with R package iGraph (v1.2.6).

Custom code used in this analysis is available at https://github.com/COG-UK/camb-uni-phylo/. Please direct further queries to the corresponding authors.

For manuscripts utilizing custom algorithms or software that are central to the research but not yet described in published literature, software must be made available to editors and reviewers. We strongly encourage code deposition in a community repository (e.g. GitHub). See the Nature Research guidelines for submitting code & software for further information.

Data

Policy information about availability of data

All manuscripts must include a data availability statement. This statement should provide the following information, where applicable:

- Accession codes, unique identifiers, or web links for publicly available datasets
- A list of figures that have associated raw data
- A description of any restrictions on data availability

The Assembled/consensus genomes generated in this study have been deposited in the GISAID database and raw reads are available from European Nucleotide Archive (ENA) under accession PRJEB37886. Pooled sample sequence raw reads and assembled sequences were deposited in the NCBI Sequence Read Archive Database (SRA; https://www.ncbi.nlm.nih.gov/sra) under the BioProject accession number PRJNA779279 (https://www.ncbi.nlm.nih.gov/bioproject/PRJNA779279). ENA and Genbank accession codes for individual sequences used in this study are available in supplementary materials. All genomes, phylogenetic trees and basic metadata are available from the COG-UK consortium website (https://www.cogconsortium.uk/data). Limited public metadata, analysis files, and processed genomic data for this work are available from GitHub at https://github.com/COG-UK/camb-uni-phylo/ (https://doi.org/10.5281/zenodo.5643354), which also contains a list of ENA and Genbank study sequence accession numbers for this study. Extended metadata are under restricted access for confidentiality reasons and in line with study ethics; requests for access should be directed to corresponding authors and specifically for Public Health England data, to the Public Health England office of data release (https://www.gov.uk/government/publications/accessing-public-health-england-data/about-the-phe-odr-and-accessing-data) with an estimated 60 working days turnaround time. Processed metadata generated for figures in this study are provided in the Source Data file.

Field-specific reporting

Please select the one below that is the best fit for your research. If you are not sure, read the appropriate sections before making your selection.

☒ Life sciences ☐ Behavioural & social sciences ☐ Ecological, evolutionary & environmental sciences

For a reference copy of the document with all sections, see nature.com/documents/nr-reporting-summary-flat.pdf

Life sciences study design

All studies must disclose on these points even when the disclosure is negative.

Sample size

The study is a descriptive epidemiological study. It is observational in nature, analyzing genomes from individuals with confirmed SARS-CoV-2 infection in a university population and its community. Data used in the study included all available genomes for these two populations. Given it is a descriptive study based on surveillance data, there were no sample size calculations conducted.

Data exclusions

The vast majority of available data was included in the study. University samples with a cycle threshold value greater than 33 were excluded due to prior data demonstrating very high failure rate of sequence generation. Sequences with higher ambiguous site content (>5%) were excluded as they can result in false inferences of genomic clustering and inaccuracies in phylogenetic tree building.

Replication

We point out this is a descriptive epidemiological study with no experiments being conducted for replication. We have however successfully repeated analysis as new data became available and with varying computational infrastructures; consistency has been demonstrated in findings by multiple phylogenetic analyses including building of a maximum likelihood tree and time-scaled trees by 4 independent researchers. For phylodynamic inferences we have conducted multiple robustness analyses in order to confirm findings. All sequencing data is publicly available to enable other researchers to replicate findings.

Randomization

This study reports genomic epidemiology derived from prospectively collected surveillance data from predefined epidemiological groups (university and community cases respectively). It does describe the impact of non-pharmacological interventions such as national lockdown

	and infection prevention control measures, however these are implemented at a national level through governmental guidelines and therefore randomization is not appropriate.
Blinding	Blinding is not applicable to this study as access to epidemiological and contact tracing data was required for inference of transmission. Further, this work was conducted with a 'real-time' with Public Health England to assist with the management of an active outbreak and inform immediate public health measures.

Reporting for specific materials, systems and methods

We require information from authors about some types of materials, experimental systems and methods used in many studies. Here, indicate whether each material, system or method listed is relevant to your study. If you are not sure if a list item applies to your research, read the appropriate section before selecting a response.

Materials & experimental systems

n/a	Involved in the study
☒	☐ Antibodies
☒	☐ Eukaryotic cell lines
☒	☐ Palaeontology and archaeology
☒	☐ Animals and other organisms
☐	☒ Human research participants
☒	☐ Clinical data
☒	☐ Dual use research of concern

Methods

n/a	Involved in the study
☒	☐ ChIP-seq
☒	☐ Flow cytometry
☒	☐ MRI-based neuroimaging

Human research participants

Policy information about studies involving human research participants

Population characteristics	665 participants were male, 690 participants were female, with data on gender not available for 99 individuals. The mean age of study cohort was 35 years (median=27, interquartile range= 19-48) years, with data on age not available for 56 individuals. In total, 972 SARS-CoV-2 cases were identified among university students and staff over the course of term (5 October to 6 December 2020). High-quality genomes were generated from 446/778 (57.3%) positive cases from the university testing programme, from 107/266 (40.2%) cases identified through the Healthcare worker (HCW) screening programme (95 HCWs, 8 students, 4 university staff) and 104 patients identified by hospital testing (71 SARS-CoV-2 positive patients from Cambridge University Hospitals (CUH) and 33 from other medical facilities in Cambridgeshire). A further 797 local cases identified by community testing during the study period were present within the COG-UK dataset, of which 17 were identified as students, 7 as university staff and 26 as HCWs. Of all identified SARS-CoV-2 cases from Cambridgeshire (university and community) during this period, 8.0% were sequenced.
Recruitment	Samples were derived from university symptomatic testing and asymptomatic COVID-19 screening programmes between October 5 2020 and December 6 2020, covering the full term. Testing for all symptomatic students and staff was available on weekdays. Screening was offered on a voluntary basis to all students residing in accommodation owned or managed by a college or the Cambridge Theological Federation. In total, 15,561 students were eligible to participate. To optimise testing efficiency, multiple swabs were pooled into the same tube of viral transport medium at the time of sample collection. Testing pools varied in size from 1 to 10 students, with each devised to include one or more student households as far as possible. In this study, households are defined as individuals who share a kitchen, bathroom and/or lounge facilities. The members of any pool testing positive were re-tested using individual confirmatory PCR tests to confirm the result and identify the positive subject(s) (see supplementary methods for further details including infection prevention control measures). Only samples from individuals that were confirmed positive upon the re-testing were used for sequencing.<br><br>SARS-CoV-2 strains circulating in the local community were identified from the COG-UK dataset for Cambridgeshire. These data were derived from local community samples from non-hospitalised, symptomatic individuals, who requested a free diagnostic test via national community testing. Other samples were derived from patients treated at three Cambridgeshire hospital trusts: Cambridge University Hospitals NHS Foundation Trust (a teaching hospital providing secondary care services for Cambridge and the surrounding area as well as tertiary referral services for the East of England and surge capacity for COVID-19); Royal Papworth Hospital NHS Foundation Trust (specialist heart and lung hospital, also providing surge capacity for COVID-19); Cambridgeshire and Peterborough NHS Foundation Trust (provider of community, mental health and learning disability services in Cambridgeshire). Hospital samples were obtained from both asymptomatic screening and those exhibiting COVID-19 symptoms. Finally, samples were derived from the asymptomatic HCW programme at Cambridge University Hospitals.<br><br>Participants were not compensated for their involvement in the study.
Ethics oversight	The COG-UK study protocol was approved by the Public Health England Research Ethics Governance Group (reference: R&D NR0195). Public Health England affiliated authors had access to identifiable Cambridgeshire community case data. This data was processed under Regulation 3 of The Health Service (Control of Patient Information) Regulations 2002- permitting the processing of confidential patient information for communicable disease and other risks to public health and as such, individual patient consent is not required. Other authors only had access to anonymised or summarised data. Ethical approval for the University of Cambridge asymptomatic COVID-19 screening programme was granted by the University of Cambridge Human Biology Research Ethics Committee (HBREC.2020.35), with informed consent gained from participants.

Note that full information on the approval of the study protocol must also be provided in the manuscript.
